# Nicotinamide nucleotide transhydrogenase deficiency and genetic susceptibility to high glucose‐mediated peritoneal injury in mice

**DOI:** 10.14814/phy2.70642

**Published:** 2025-11-14

**Authors:** Margarete C. Ohse, Song Rong, Sonja Schmidt, Michael S. Balzer, Christoph Nikolin, Sibylle von Vietinghoff, Hermann Haller, Kai M. Schmidt‐Ott, Yulia Kiyan, Nelli Shushakova

**Affiliations:** ^1^ Department of Nephrology and Hypertension Hannover Medical School Hannover Germany; ^2^ Phenos GmbH Hannover Germany; ^3^ Medical Department, Division of Nephrology and Internal Intensive Care Medicine Charité Universitätsmedizin Berlin Berlin Germany; ^4^ Institute of Transfusion Medicine and Transplant Engineering Hannover Medical School Hannover Germany; ^5^ Nephrology Section, Medical Clinic 1 University Hospital Bonn and Bonn University Bonn Germany; ^6^ Mount Desert Island Biological Laboratory Salsbury Cove Maine USA

**Keywords:** C57BL6 mice, nicotinamide nucleotide transhydrogenase, NNT, oxidative stress, peritoneal dialysis

## Abstract

The genetic predisposition to high glucose‐induced peritoneal membrane (PM) injury during peritoneal dialysis (PD) and its mechanisms are of substantial clinical interest. We compared PD‐induced peritoneal injury between two closely related mouse substrains, C57BL/6J and C57BL/6N, which differ in the function of the mitochondrial enzyme nicotinamide nucleotide transhydrogenase (NNT). *Nnt*(+/+) C57BL/6N mice exhibited significantly greater susceptibility, as indicated by mesothelial cell loss, fibrosis, neoangiogenesis, inflammation, M1 macrophage infiltration, and reduced ultrafiltration. To further investigate NNT's role, we silenced NNT in vitro. Knockdown prevented mitochondrial ROS accumulation, reduced pro‐inflammatory mediator release in mesothelial cells, inhibited M1 polarization in macrophages, and impaired fibroblast proliferation under high glucose. We also observed a reverse NNT reaction in fibroblasts, contributing to glucose‐induced ROS. Our findings indicate reduced genetic susceptibility of *Nnt*(−/−) C57BL/6J mice to PD‐induced PM damage and identify NNT as a potential therapeutic target for PD‐associated peritoneal injury.

## Graphical abstract

High‐glucose peritoneal dialysis (PD) fluid induces oxidative stress, inflammation, and fibrosis in the peritoneum. In vivo, Nnt‐deficient C57BL/6J mice showed significantly reduced PD‐induced peritoneal injury, inflammation, and fibrosis compared with Nnt‐competent C57BL/6N controls. Complementary in vitro NNT‐silencing experiments in peritoneal mesothelial cells, macrophages, and fibroblasts demonstrated reduced high‐glucose‐induced ROS generation, M1 polarization, and profibrotic signaling. Together, these data identify NNT as a key regulator of mitochondrial redox balance that may switch to a pro‐oxidative “reverse mode” under high‐glucose conditions, thereby representing a potential therapeutic target to prevent PD‐associated peritoneal damage.

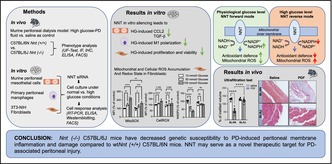

## 1 INTRODUCTION

Peritoneal dialysis (PD) is an established, cost‐effective therapy for end‐stage renal disease (Davies, 2013; Perl & Bargman, 2009; Thurlow et al., 2021). However, high glucose (HG)‐containing PD fluid (PDF), with glucose concentrations up to 40‐fold higher than those in plasma, metabolically reprograms peritoneal cells through a process termed hyperglycolysis (Huang et al., 2023; Masola et al., 2022; Si et al., 2019), which induces oxidative stress in the peritoneal cavity (Ha & Lee, 2000; Ksiazek et al., 2007; Roumeliotis et al., 2020) and thereby impacts mitochondria (Ishibashi et al., 2002). Reactive oxygen species (ROS)‐dependent mitochondrial dysfunction and oxidative stress have been demonstrated to contribute to deleterious peritoneal changes (Helmke et al., 2021; Hung et al., 2014; Ramil‐Gomez et al., 2021; Ramil‐Gomez et al., 2022), including peritoneal inflammation, severe submesothelial extracellular matrix (ECM) deposition, and neovascularization, ultimately leading to adverse clinical outcomes in PD patients (Yoon et al., 2016).

Mouse PD models are critical tools for testing newly developed PDFs and for investigating the contributions of specific genes in PD‐induced peritoneal membrane damage. However, genetic differences between mouse strains can substantially impact fibrogenic responses, one of the most important endpoints in PD studies (Margetts et al., 2013; Wang et al., 2016). Two commonly used mouse substrains, C57BL/6J and C57BL/6N, are derived from the same parental C57BL/6 strain but exhibit distinct genotypic differences, leading to notable phenotypic variations, especially in metabolic research models (Fontaine & Davis, 2016; Mekada & Yoshiki, 2021). In the context of PD, a spontaneous loss‐of‐function deletion (exon 7–11) within the mitochondrial *Nnt* gene, observed exclusively in C57BL/6J mice (Mekada et al., 2009; Simon et al., 2013), is of particular interest.

*Nnt* encodes nicotinamide nucleotide transhydrogenase (NNT), a nuclear‐encoded protein within the inner mitochondrial membrane. Under aerobic conditions, NNT maintains a high mitochondrial NADPH/NADP^+^ ratio via catalyzing the transfer of hydrogen from NADH to NADP^+^ by oxidation and reduction (Ho et al., 2017; Kampjut & Sazanov, 2019; Nesci et al., 2020), serving as the primary source of mitochondrial NADPH (Rydstrom, 2006). This process, driven by proton translocation across the inner membrane, makes NNT a proton motive force‐dependent enzyme (Ronchi et al., 2016). Since peroxide detoxification is mainly driven by NADPH, NNT has long been considered a key antioxidant mitochondrial enzyme (Meimaridou et al., 2018; Ronchi et al., 2016; Yin et al., 2012). Loss of NNT activity results in mitochondrial dysfunction and promotes chronic inflammation associated with metabolic diseases, including diabetes (Regan et al., 2022). For instance, C57BL/6J mice exhibit impaired glucose tolerance due to glucose‐induced oxidative stress and decreased ATP synthesis in pancreatic beta cells associated with the *Nnt* mutation (Toye et al., 2005). Furthermore, transgenic expression of the functional *Nnt* gene in C57BL/6J‐background mice resulted in improved glucose tolerance (Freeman et al., 2006; Kierans & Taylor, 2021). In inbred strains of diabetes‐susceptible DBA/2 x C57BL/6 backcrossed mice, a positive correlation between insulin secretion and NNT activity has been observed (Aston‐Mourney et al., 2007).

Under conditions of limited mitochondrial respiratory substrates, a decreased electrical potential across the inner mitochondrial membrane (ΔΨ) and a more oxidized NAD^+^ redox potential shift the net flux of the NNT reaction toward NAD^+^ reduction and NADPH oxidation (i.e., the reverse reaction) (Francisco et al., 2022). This reversal sustains NADH and ATP production, resulting in NADPH oxidation at the expense of mitochondrial antioxidative capacity. This switch of NNT activity to a pro‐oxidative reverse mode has been shown in vitro for human cystic fibrosis epithelial cells (Favia & Atlante, 2021) and mouse pancreatic islet cells (Santos et al., 2017). This pro‐oxidative reverse NNT reaction has also been demonstrated in vivo in mouse hearts under mechanical overload (Nickel et al., 2015), leading to NADPH depletion, increased mitochondrial ROS emission, and maladaptive cardiac remodeling in C57BL/6N mice. Moreover, C57BL/6J mice subjected to postnatal hypoxia–ischemia demonstrated reduced brain damage, and the authors postulated the NNT reverse mode as a possible explanation (Wolf et al., 2016).

The precise genetic mechanisms of PD‐associated membrane failure and the role of NNT in the peritoneal response to PDF are currently unknown. However, given the critical role of NNT in mitochondrial redox homeostasis and cellular antioxidant defense (Yin et al., 2012), both of which are severely challenged during PD (Ramil‐Gomez et al., 2021; Ramil‐Gomez et al., 2022), we hypothesized that NNT might either protect peritoneal cells from HG‐induced ROS generation and mitochondrial dysfunction or exacerbate HG‐induced peritoneal damage if it acts in reverse mode.

To investigate this, we first compared C57BL/6J and C57BL/6N mice in a PD mouse model and found that, in contrast to C57BL/6N, C57BL/6J mice were nearly completely protected from PD‐induced PM damage. To further examine the potential role of NNT in PD‐induced PM damage and minimize effects from other genetic differences, we conducted in vitro experiments using NNT‐specific small interfering RNA (siRNA) in mouse peritoneal mesothelial cells, primary peritoneal macrophages, and NIH‐3T3 fibroblasts. The results of these in vitro experiments mirrored our in vivo findings, suggesting that at least part of the PD‐induced detrimental peritoneal changes in C57BL/6N mice may be mediated by NNT.

## 2 METHODS

### 2.1 Animals

Twelve‐week‐old female C57BL/6N and C57BL/6J mice (Charles River Laboratories, Sulzfeld, Germany) were housed in individually ventilated cages with additional nesting material as environmental enrichment under pathogen‐free laboratory conditions (23°C, 12:12 h dark/light cycle) at the animal facility of Phenos GmbH (Hannover, Germany). The mice were acclimated for 3 weeks before the experiments and had free access to a standard diet (Altromin Int, Germany, 1324) and tap water. NNT was genotyped via allele‐specific RT‐PCR (ASQ) (primers from Eurofins Scientific SE, Luxembourg, see Supplementary File, Table S1 for primer sequences), as described in Protocol 26,539 on the Jackson Laboratory website. Genotyping by PCR confirmed *Nnt*(+/+) genotype for C57BL/6N mice and *Nnt*(−/−) genotype for C57BL/6J mice.

### 2.2 Murine peritoneal dialysis model

The Department for Food Safety and Animal Welfare of Lower Saxony, LAVES, Germany (approval number: 33.19‐42502‐04‐16/2266) permitted the experiments.

Mice were randomly assigned to experimental groups using a computer‐generated sequence (G*Power 3.1) and subjected to chronic PDF exposure or isotonic 0.9% saline as control for 5 weeks as described previously (Wang et al., 2016). Briefly, via an intraabdominal catheter, connected to a subcutaneous mini‐access port (Access Technologies, Skokie, IL, USA, MMP‐SIL‐C03), 1 week of daily 0.2 mL saline injections was followed by 5 weeks of daily 1.5 mL injections of either conventional lactate‐buffered, 4.25% glucosePDF (CAPD/DPCA3 StaySafe, Fresenius Medical Care, Bad Homburg, Germany) or isotonic 0.9% saline as control. The surgeries were performed under appropriate anesthesia (isoflurane) along with pre‐ and intra‐operative analgesia (Turbogesic® (Butorphanol) 1 mg/kg s.c.) to minimize pain. Metamizole (200 mg/kg p.o.) was administered for post‐operative analgesia for 3 days following surgery. A total of 34 mice were used in the experiment. The groups comprised 6 animals for the saline treatment and 11 for the PDF treatment. Sample sizes were calculated using G*Power 3.1 based on previous studies, aiming for a statistical power of 0.8 and an α‐level of 0.05 for the primary endpoint parameters, the UF test, peritoneal fibrosis and inflammation. The order of daily cage processing and the treatment of individual mice were randomized and counterbalanced. During the experiment, the health of the mice was checked daily. The following exclusion criteria, which also served as humane endpoints, were applied: more than 20% weight loss, any signs of port infection, or abnormal activity. No adverse events were observed during experiments. No animals were excluded from the experiment and all data points were included in the analysis. On the last day of treatment, peritoneal function was assessed by an ultrafiltration test. For this purpose, PDF was instilled in all animals, and after 90 min the mice were euthanized by isoflurane overdose and the intraabdominal fluids were completely drained. Ultrafiltration capacity was calculated as the x‐fold increase of collected volume compared to instilled volume. The collected lavage fluids were used for the FACS analysis of the peritoneal influx of inflammatory cells and for the measurements of inflammatory mediators. The parietal peritoneum was acquired for histological analysis. Investigators performing outcome assessments were blinded to group allocations.

### 2.3 Analysis of inflammatory cells and cytokines in peritoneal effluents

Inflammatory cell populations in peritoneal effluents were analyzed by flow cytometry using a fluorescence‐activated cell sorter (FACS) Canto II cytometer (BD Biosciences, Heidelberg, Germany). The following monoclonal antibodies (BioLegend, San Diego, CA, USA) were used to detect myeloid cells, polymorphonuclear leukocytes, macrophages, T cells, and B cells in peritoneal effluents respectively: anti‐CD11b (clone M1/70, 101207), anti‐Gr1 (clone RB6‐8C5, 108407), anti‐F4/80 (clone BM8, 123109), anti‐TCRb (clone H57‐597, 109201), and anti‐CD19 (clone 6D5, 115501). Data analysis was performed by FlowJo software (Tree Star Inc., Ashland, OR, USA).

Analysis of TNF‐α, CCL2, IL‐6 and IL‐10 was accomplished by FACS using a cytometric bead array assay (Mouse Inflammation kit, BD Biosciences, San Jose, CA, USA, 552364) according to the manufacturer's instructions. Measurements of TGF‐β, VEGF‐A/B and macrophage inflammatory protein‐2 (CXCL2) were conducted by ELISA (R&D Systems, Minneapolis, MN, USA, DY1679, DY493, DY453 for TGF‐β, VEGF‐A/B, and CXCL2, respectively).

### 2.4 Histochemical and immunofluorescence analysis of peritoneal tissue

Peritoneal specimens were fixed in 4% formaldehyde for 24 h, followed by dehydration in an ascending ethanol series and paraffin embedding. Light and fluorescence microscopy were performed on 2.5 μm thick tissue sections via a Keyence BZ‐9000 fluorescence microscope (Keyence Corporation, Osaka, Japan) with a Nikon CFI 60 Series infinite optical system. All images were taken at 200× magnification. The submesothelial layer thickness of the peritoneum was determined via Masson's trichrome staining (Sigma‐Aldrich, HT15) by applying 50 equidistant measurements (from the exterior mesothelial surface to the top of the upper muscular layer) per animal and expressed as the mean value.

Picrosirius red staining (Sigma‐Aldrich, P6744, 365548) was conducted for the analysis of collagen I and III. The percentage of collagen I and III positive area was assessed using ImageJ software (NIH, Bethesda, MD, USA, version 1.53) in 10 adjacent high magnification images representing a total mesothelial length of 5.0–5.5 mm with 1 picture accounting for 500–600 μm of mesothelial length. A submesothelial band of 250 μm in width measured perpendicularly to the mesothelial layer was analyzed for fibrosis using Volker Baecker's MRI fibrosis quantification macro for ImageJ (http://dev.mri.cnrs.fr/projects/imagej‐macros/wiki/Fibrosis_Tool).

Immunofluorescence analysis of peritoneum was performed for CD31 (Dianova, Hamburg, Germany, clone SZ31, DIA‐310), F4/80 (Santa Cruz Technology, clone BM‐8, sc‐52664), CD38 (Santa Cruz Technology, clone H‐11, sc‐374650), CD206 (Bioss Antibodies, Woburn, MA, USA, rabbit polyclonal, bs‐4727R), α‐SMA (Sigma‐Aldrich, clone 1A4, A5228), pan‐cytokeratin (Santa Cruz Technology, sc‐15367), and NNT (Santa Cruz Technology, sc‐390236). For fluorescent visualization of bound primary antibodies, Alexa555 or Alexa488 conjugated secondary antibodies (Cell Signaling Technology, Danvers, MA, USA, 4409, 4417, 4408, 4412) were used. Background control staining performed by incubating with secondary antibodies alone proved to be negative. The slides were also stained with Lycopersicon esculentum (Tomato) lectin (Vector Laboratories, Newark, CA, USA, DL‐1174‐1).

### 2.5 Cells and cell culture conditions

For in vitro experiments, we analyzed three different cell types: immortalized murine peritoneal mesothelial cells (MPMC), primary peritoneal macrophages (PMΦ) isolated from untreated female C57BL/6N and C57BL/6J mice, and immortalized murine NIH‐3T3 fibroblasts (ATCC, Manassas, VA, USA, CRL‐1658).

Immortalized MPMCs were generated as described previously (Wang et al., 2016). They were grown to 80% confluency in RPMI‐1640 (Sigma‐Aldrich, R5886) cell culture medium supplemented with 10% fetal calf serum (FCS), 1% insulin/transferrin/selenium A, 1% penicillin–streptomycin (all from Life Technologies, Carlsbad, CA, USA) and 0.4 mg/mL hydrocortisone (Sigma‐Aldrich, H6909) under permissive conditions at 33°C, 5% CO2, and 95% humidity, followed by cell differentiation at non‐permissive temperature of 37°C for 72 h.

Murine NIH‐3 T3 fibroblasts were grown to 80% confluence in DMEM (Thermo Fisher Scientific, Waltham, MA, USA, 15235697) containing 10% FCS, 100 U/mL penicillin, and 0.1 mg/mL streptomycin at 37°C, 5% CO_2_, and 95% humidity.

Primary PMΦ were isolated from untreated female C57BL/6N and C57BL/6J mice by peritoneal lavage and re‐suspended in RPMI‐1640 medium supplemented with 10% FCS, 100 U/mL penicillin, and 0.1 mg/mL streptomycin. The culture plates were incubated overnight at 37°C, 5% CO_2_, and 95% humidity, to allow macrophage adherence. Non‐adherent cells were removed by vigorous washing with RPMI‐1640 medium.

### 2.6 Nnt silencing in vitro

Cells were cultured under normal glucose (NG, 10 mmol/L) or high glucose (HG, 25, 50, or 100 mmol/L) conditions. NNT expression was evaluated by RT‐PCR and Western blotting after 24 and 48 h, respectively.

NNT silencing experiments were conducted using NNT‐specific siRNA (Santa Cruz Biotechnology, sc‐150013), and JetPRIME transfection agent (Polyplus, Illkirch, France, 101000046), following the manufacturer's instructions. Cells treated only with the transfection reagent (mock), and untreated cells served as controls. The effects of NNT silencing on HG‐induced cellular responses, such as pro‐inflammatory mediator release, expression of α‐SMA, cell viability, proliferation, M1/M2 macrophage polarization, NADPH/NADP^+^ ratio, and ROS accumulation, were then investigated.

### 2.7 Stimulation of MPMC with HG and TGF‐β

MPMCs were starved overnight in 1% FCS/RPMI 1640 medium and then stimulated with medium containing NG (10 mmol/L) or HG (50 or 100 mmol/L) or 10 ng/mL recombinant TGF‐β. CCL2 and TGF‐β were analyzed in conditioned medium by ELISAs (R&D Systems, DY479, DY1679) after 24 and 72 h, respectively. α‐SMA expression used as a marker for MMT was evaluated by RT‐PCR and western blot analysis.

### 2.8 Stimulation of PMΦ with HG and LPS

Naïve PMΦ were cultured under NG (10 mmol/L) or HG (50 or 100 mmol/L) for 48 h, and subsequently stimulated with 100 ng/mL LPS for 6 h. The levels of inflammatory cytokines TNF‐α, CCL2, IL‐6, and IL‐10 in conditioned medium were measured by FACS using a cytometric bead array assay as described for peritoneal effluents.

### 2.9 Stimulation of 3T3 fibroblasts with HG

Starved 3T3 fibroblasts were cultured under NG (10 mmol/L) or HG (50 or 100 mmol/L) for 24 h and thereafter HG‐induced mitochondrial and cellular ROS accumulation was evaluated by FACS analysis using MitoSOX™ red (Thermo Fisher Scientific, Waltham, MA, USA, M36007) and CellROX™ green kits (Thermo Fisher Scientific, Waltham, MA, USA, C10492) staining as described by the manufacturer. In another set of experiments, the NADPH/NADP^+^ ratio was measured and calculated by the use of the NADP/NADPH‐Glo Assay (Promega, Fitchburg, WI, USA, G9081), following the manufacturer's instructions. The proliferation rate was measured after incubating cells in ascending glucose concentrations in the presence of 10% FCS for 24 h using the CCK‐8 kit (Sigma‐Aldrich, 96992), regarding the manufacturer's instructions. Glucotoxicity was evaluated after arresting cell proliferation by starving cells in 1% FCS for 24 h, followed by treatment with varying glucose concentrations. Cell viability was measured by the use of the CCK‐8 assay.

### 2.10 RNA isolation, real time polymerase chain reaction and western blot analysis

Total RNA was extracted by the use of the RNeasy mini kit (Qiagen, Venlo, The Netherlands, 74106) and reverse transcription was performed using the Transcriptor First Strand cDNA Synthesis Kit (Roche Diagnostics, Penzberg, Germany, 04897030001). Amplified cDNA was used for RT‐PCR analysis of NNT (primer from Biomol, Hamburg, Germany, VMPS‐4376) and α‐SMA mRNA expression (primer from Qiagen, QT00140119), as well as CCL2 mRNA expression (primer from Eurofins; see Supplementary File, Table S1 for primer sequences). Results were normalized to HPRT‐1 mRNA expression (Qiagen, QT00166768). RT‐PCR was accomplished in triplicates via the SYBR green technique (TB Green Premix Ex Taq, Tli RNase H Plus, TaKaRa Bio Inc., Shiga, Japan, RR420WR) on a LightCycler480 Instrument (Roche Diagnostics). Western blotting analysis was carried out as SDS‐PAGE according to standard protocols. Briefly, 60 μg of protein was loaded on a polyacrylamide gel. PVDF membranes (Sigma‐Aldrich, 03010040001) were blocked with 3% BSA, and probed with primary antibodies against NNT (Santa Cruz Technology, sc‐390236), α‐SMA (Sigma‐Aldrich, A5228), GAPDH (Sigma‐Aldrich, AB2302) and β‐tubulin (Invitrogen, Thermo Fisher Scientific, Waltham, MA, USA PA1‐41331). Visualization was accomplished by horseradish peroxidase‐labeled secondary antibodies (Cell Signaling 7076S and 7074S) and the VersaDoc gel imaging system and finally quantified using Quantity One Software (both by BioRad Laboratories, Hercules, CA, USA). Uncropped western blot images are shown in the Supplementary File.

### 2.11 Statistical analysis

Data are reported as means ± SD, if not indicated otherwise. The D'Agostino and Pearson omnibus normality test was used to test for normality. Multiple comparisons were analyzed by one‐way analysis of variance with Sidak's post hoc correction or the Kruskal–Wallis nonparametric test with Dunn's post hoc correction. *p*‐values <0.05 were considered statistically significant. GraphPad Prism 9 (GraphPad Software, San Diego, CA, USA) was used for data analysis.

## 3 RESULTS

### 3.1 Peritoneal dialysis induces severe functional and structural changes of peritoneal membrane in C57BL/6N mice, but not in C57BL/6J mice

**FIGURE 1:**
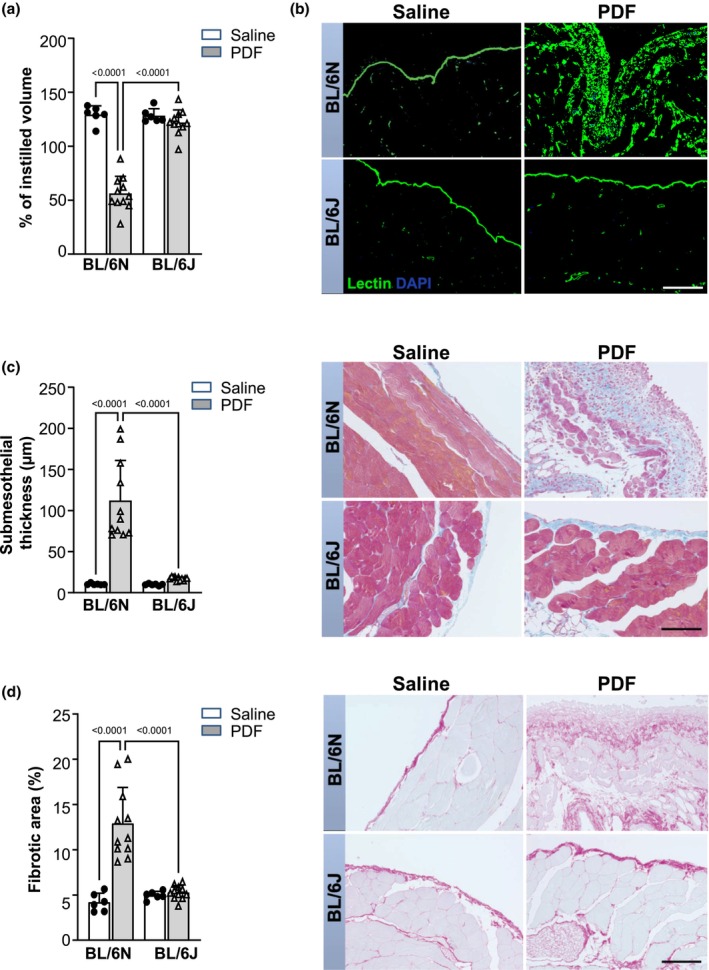
Functional and structural impairment after 5 weeks of daily treatment with 4.25% glucose PDF is severe in C57BL/6N mice, but mild in C57BL/6J mice. Mice were instilled with saline (*n* = 6) or with PDF (*n* = 11) for 5 weeks. (a) An ultrafiltration test was performed on the last day of treatment. Ultrafiltration capacity was quantified as the percentage of peritoneal effluent recovered 90 min after PDF instillation. (b) Representative images of tomato lectin staining of the peritoneum visualize pronounced structural chances of the peritoneal membrane in PDF‐instilled C57BL/6N, but not C57BL /6J mice. Scale bar = 100 μm. (c) Representative images of Masson trichrome stain demonstrate increased deposition of extracellular matrix, cell numbers, and thickness of PM in PD‐treated C57BL/6N, but not in C57BL/6J mice. Scale bar = 100 μm. Graph represents quantification of PM thickness. (d) Representative images of Picrosirius red staining, indicating peritoneal collagen deposition, and associated analysis of percentage of positive area. Severe submesothelial fibrosis was only detected in PD‐treated C57BL/6N mice. Scale bar = 100 μm. Data are presented as mean ± SD.

After 5 weeks of PD, peritoneal ultrafiltration capacity was markedly decreased in PDF‐instilled C57BL/6N animals, while it was preserved in C57BL/6J mice. In both sub‐strains, saline treatment did not lead to any significant alterations in peritoneal ultrafiltration capacity (Figure 1a). These functional differences were mirrored by morphological changes. *Lycopersicon esculentum* (tomato) lectin was used as a marker for glycocalyx on peritoneal mesothelium, infiltrating leukocytes, and endothelial cells. As expected, in saline‐treated mice of both sub‐strains, a positive signal was observed only on mesothelium and the endothelium of blood vessels located between muscles. In PDF‐treated mice, loss of the mesothelial monolayer and submesothelial accumulation of lectin‐positive cells, as well as new vessel formation, was observed only in C57BL/6N, but not in C57BL/6J mice (Figure 1b). Structural peritoneal changes were further evaluated by Masson's trichrome staining. We found pronounced peritoneal thickening, due to ECM deposition and hypercellularity in C57BL/6N mice after PD treatment. By contrast, PDF‐instilled C57BL/6J mice scarcely displayed peritoneal structural changes (Figure 1c).

PD‐induced changes in collagen deposition were further evaluated by Picrosirius red staining. A strong increase in the fibrotic area was observed only in C57BL/6N after PD treatment, but not in C57BL/6J mice (Figure 1d).

**FIGURE 2:**
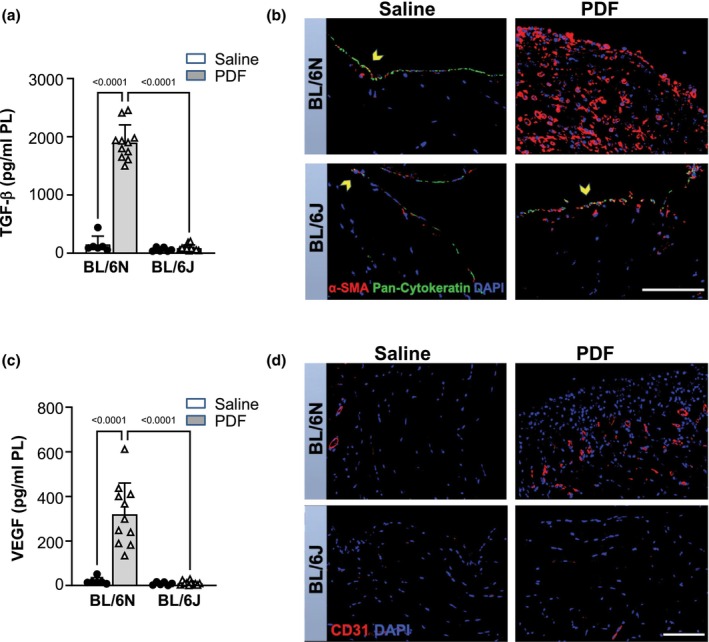
PD‐induced and TGF‐β‐mediated transformation of mesothelial cells and accumulation of myofibroblasts, and peritoneal new vessel formation are significantly increased in C57BL/6N, but not in C57BL/6J mice. Mice were instilled with saline (*n* = 6) or with PDF (*n* = 11) for 5 weeks. (a) Analysis of pro‐fibrogenic mediator TGF‐β in peritoneal effluents, analyzed by ELISA. (b) Immunofluorescence staining for α‐SMA (red) and pan‐cytokeratin (green), representative images. PD‐treated C57BL/6N mice exhibited submesothelial accumulation of α‐SMA‐positive cells, mesothelial denudation and complete loss of pan‐cytokeratin, indicating mesothelial‐to‐mesenchymal‐transition (MMT). In contrast, the mesothelial monolayer in C57BL/6J mice showed positive staining for both markers, with only few submesothelial α‐SMA‐positive cells observed, suggesting incipient MMT. Scale bar = 100 μm. (c) Quantification of VEGF in peritoneal effluents via ELISA demonstrates significantly enhanced levels only in C57BL/6N mice. (d) Representative images of immunofluorescence staining with anti‐CD31 display markedly pronounced microvessel density only in PD‐treated C57BL/6N mice. Scale bar = 100 μm. Data are presented as mean ± SD.

In line with pronounced fibrosis in PDF‐instilled C57BL/6N mice, a strong up‐regulation of the fibrogenic mediator TGF‐β was detected only in the effluents of these animals, but not in the effluents of PDF‐instilled C57BL/6J mice and in saline‐treated controls (Figure 2a). Additionally, we evaluated PD‐induced mesothelial‐to‐mesenchymal transition (MMT), primarily mediated by TGF‐β, and the accumulation of myofibroblasts by immunohistochemistry. Alpha smooth muscle actin (α‐SMA) was used as a myofibroblastic marker and pan‐cytokeratin as a marker for peritoneal mesothelium. Double staining for α‐SMA/pan‐cytokeratin revealed severe peritoneal accumulation of α‐SMA‐expressing cells, which were completely negative for the mesothelial marker pan‐cytokeratin in PDF‐instilled C57BL/6N mice (Figure 2b). This indicates not only myofibroblast accumulation but also the loss of the mesothelial cell monolayer on the peritoneal surface. In contrast, an intact mesothelial monolayer was observed in PDF‐treated C57BL/6J mice and both control groups. In C57BL/6J mice, co‐expression of α‐SMA and pan‐cytokeratin was detected within the upper mesothelial monolayer, presumably as part of incipient mesothelial alterations due to treatment. Similar results, but with a weaker signal for α‐SMA, were found in both control groups.

Enlargement of the peritoneal vessel surface area, which contributes to ultrafiltration failure, is one major reason for PD failure (Shi et al., 2022). Analysis of peritoneal effluents revealed a significantly elevated concentration of VEGF, a marker for neoangiogenesis, in samples from PDF‐instilled C57BL/6N compared to C57BL/6J mice and both control groups (Figure 2c). Additionally, peritoneum samples were stained for CD31, a marker for endothelial cells. PDF exposure resulted in markedly pronounced new vessel formation within the peritoneum of C57BL/6N mice, but not in C57BL/6J and control saline‐treated mice (Figure 2d). These results were consistent with the formation of new vessels in the peritoneum of C57BL/6N mice as detected by lectin staining (see above, Figure 1b).

### 3.2 PD treatment induces a strong inflammatory response and M1 macrophage polarization in C57BL/6N, but not in C57BL/6J mice

**FIGURE 3:**
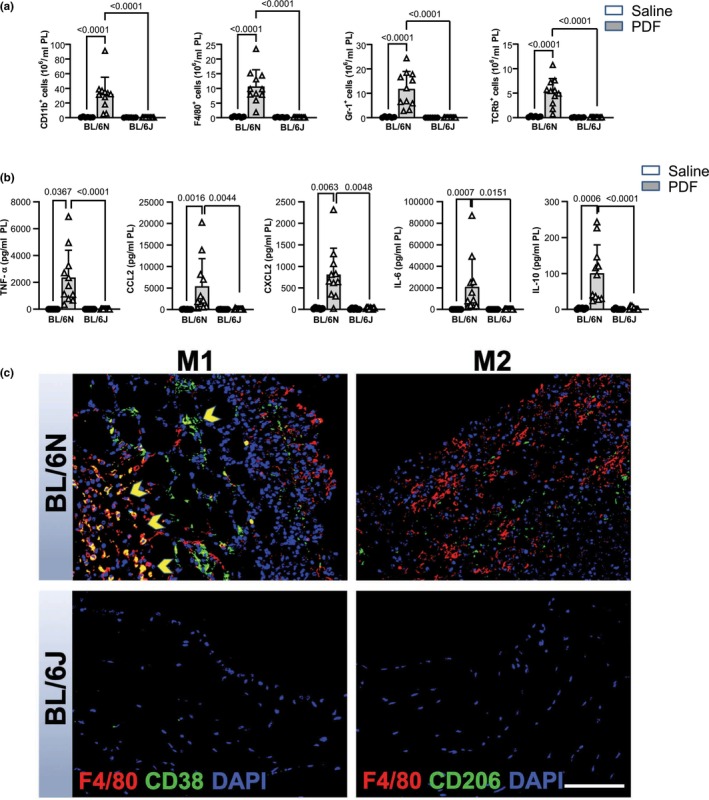
PD‐induced peritoneal inflammation in C57BL/6N mice is significantly increased compared to C57BL/6J mice. Mice were instilled with saline (*n* = 6) or with PDF (*n* = 11) for 5 weeks. (a) Inflammatory cell populations in peritoneal effluents. FACS analysis was performed for leukocytes (CD11b^+^), T cells (TCR‐β^+^), macrophages (F4/80^+^) and polymononuclear neutrophils (PMN) (Gr1^+^). (b) Inflammatory cytokines in peritoneal effluents. TNF‐α, CCL2, IL‐6 and IL‐10 were measured via bead‐based flow cytometry, CXCL2 chemokine was analyzed via ELISA. (c) M1/M2 macrophage polarization in PD‐treated mice. Double‐staining of F4/80 and CD38, as well as F4/80 and CD206. Representative images show submesothelial invasion of F4/80^+^ and CD38^+^ macrophages (indicated by yellow arrows) in C57BL/6N mice, indicating predominance of the pro‐inflammatory macrophage subtype M1. No positive staining was detected in control mice (not shown). Scale bar = 100 μm. Data are presented as mean ± SD.

We further investigated the extent of peritoneal inflammation and again, we observed distinctly divergent results between both substrains. Peritoneal lavage of PDF‐treated C57BL/6N mice exhibited a strong influx of CD11^+^ inflammatory cells, macrophages, polymorphonuclear neutrophils (PMN), and T cells. This influx was observed neither in effluents of C57BL/6J mice nor in saline‐treated controls (Figure 3a). Consistent with this, the levels of pro‐inflammatory mediators TNF‐α, IL‐6, CCL2, CXCL2, as well as the anti‐inflammatory IL‐10 in effluents, demonstrated a strong increase in PDF‐treated C57BL/6N mice compared to the saline group. Only a moderate and non‐significant elevation of IL‐6, CCL2 and CXCL2, and no increase of IL‐10, was observed in effluents of PDF‐treated C57BL/6J mice (Figure 3b). Immunofluorescence staining of peritoneal tissue sections of C57BL/6N mice revealed a pronounced positive signal of F4/80 and CD38 co‐expressing cells, indicating peritoneal infiltration of pro‐inflammatory M1 macrophages. M2 macrophages, identified by dual positivity for markers F4/80 and CD206, were sparsely detected. Underlining previous results, F4/80+ macrophages were rarely observed in samples from C57BL/6J, and no positive signal was detected for CD38 or CD206 (Figure 3c).

Taken together, we demonstrate that only C57BL/6N, but not C57BL/6J, mice are susceptible to PD‐induced peritoneal membrane damage.

### 3.3 NNT expression is increased under HG conditions in MPMC and in NIH‐3T3 fibroblasts, but not in PMΦ

**FIGURE 4:**
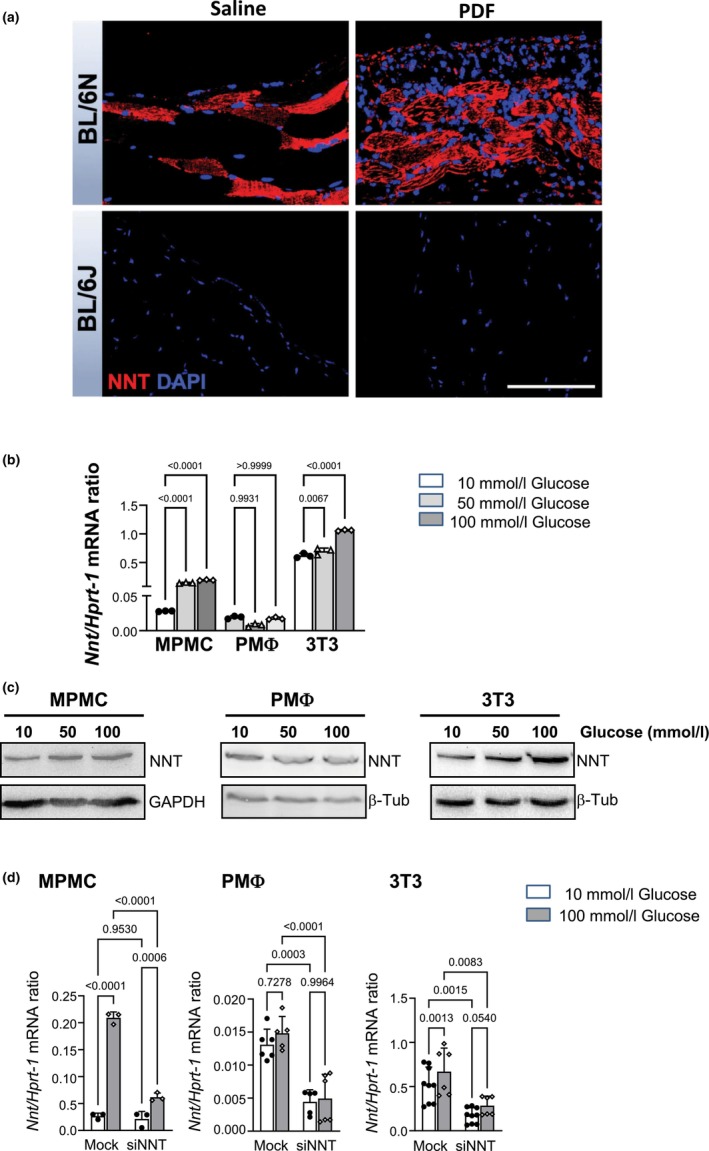
Expression of NNT is up‐regulated under HG condition in vitro in murine peritoneal mesothelial cells, primary peritoneal macrophages, and NIH‐3T3 fibroblasts and in vivo in the submesothelial space of PD‐treated BL6N mice. (a) Mice were instilled with saline (*n* = 6) or with PDF (*n* = 11) for 5 weeks. Representative images of NNT staining of the peritoneum demonstrate increased submesothelial NNT positivity in PDF‐instilled C57BL/6N mice. Scale bar = 100 μm. (b) mRNA NNT expression after 24 h of cell incubation under NG or HG conditions. (c) Western blot analysis for NNT protein expression demonstrates increased levels in MPMC and NIH‐3T3 fibroblasts, but not in PMΦ after 48 h of incubation with HG. Representative results of 3 independent experiments are shown. For uncropped gels, see Supplementary File, Figure 1. (d) Effective NNT silencing in all three cell types after 24 h of cultivation under NG/HG conditions. Data are presented as means ± SD from at least three independent experiments performed in triplicate for each cell type/conditions.

C57BL/6N and C57BL/6J substrains exhibit several genetic variations (Mekada & Yoshiki, 2021; Simon et al., 2013). Previously, a spontaneous loss‐of‐function deletion (exon 7–11) within the mitochondrial *Nnt* gene, had been reported in C57BL/6J mice (Mekada et al., 2009; Simon et al., 2013). This prompted us to explore a potential involvement of NNT in the observed differential responses of C57BL/6N‐ and C57BL/6J substrains to PD. We observed marked NNT positivity in the peritoneum of PD‐treated, but not in saline‐treated C57BL/6N mice (Figure 4a). NNT staining was also positive in striated muscle cells underlying the peritoneum, serving as an internal positive control. In contrast, NNT protein staining was entirely absent in C57BL/6J mice, both in the peritoneum and in striated muscle cells, confirming the complete loss of NNT protein in this strain (Figure 4a). We then evaluated NNT expression in mouse MPMC, PMΦ and NIH‐3 T3 fibroblasts cultured under normal (NG, 10 mmol/L) or high glucose (HG, 50 mmol/L and 100 mmol/L) conditions for 24 and 48 h, respectively, using RT‐PCR (Figure 4b) and Western blotting (Figure 4c). The highest NNT expression was observed in NIH‐3T3 fibroblasts. HG exposure caused increased NNT expression in a dose‐dependent manner at both the mRNA and protein levels in MPMC and NIH‐3T3 fibroblasts compared to cells cultured under NG, but had no effect in primary PMΦ. NNT expression could be successfully knocked down using NNT siRNA technology in all three cell types (Figure 4d).

### 3.4 NNT mediates HG‐induced CCL2 and TGF‐β release in MPMC, but has no effect on mesothelial‐to‐mesenchymal‐transition (MMT)

**FIGURE 5:**
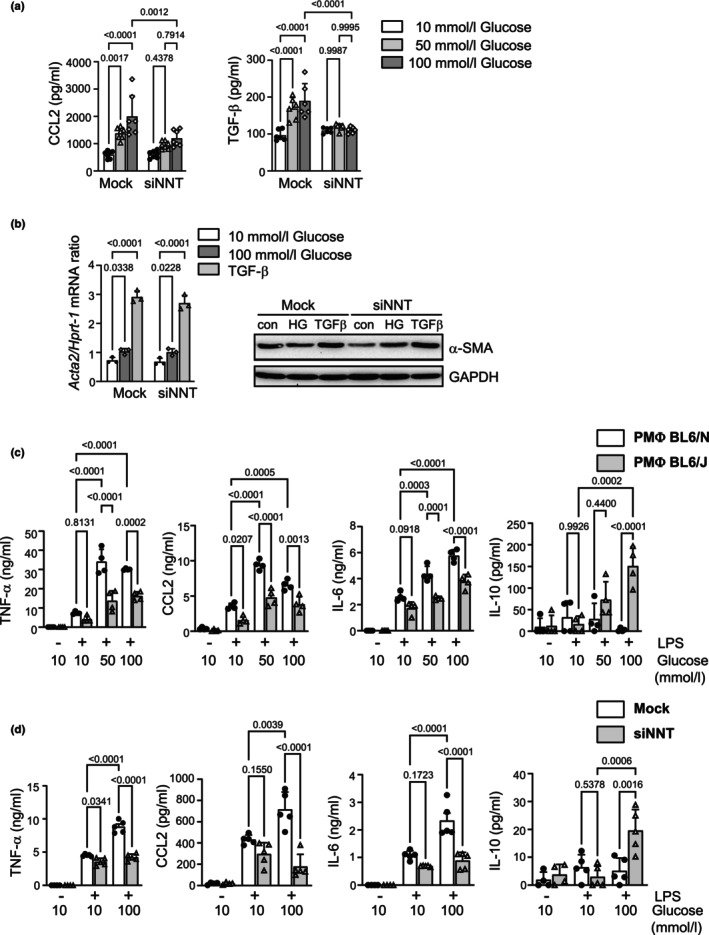
Effects of NNT silencing on HG‐induced responses in murine peritoneal mesothelial cells (MPMC) and primary peritoneal macrophages. (a) Impact of NNT silencing on the release of inflammatory CCL2 and pro‐fibrogenic TGF‐β from MPMC was assessed after cultivation under NG/HG conditions. Conditioned medium was analyzed by ELISA after 24 and 72 h, respectively. NNT silencing significantly reduced the release of CCL2 and TGF‐β under HG conditions. (b) Expression of α‐SMA was measured by RT‐PCR and Western blot analysis after treatment with different glucose concentrations and stimulation with 10 ng/mL TGF‐β. NNT silencing did not affect the α‐SMA expression at either mRNA or protein level. For uncropped gels, see Supplementary File, Figure 2. Data are presented as means ± SD from at least three independent experiments performed in triplicate for each readout/condition. (c) Comparison of naïve PMΦ from C57BL/6N and C57BL/6J mice cultured under NG or HG for 48 h, followed by stimulation with 100 ng/mL lipopolysaccharides (LPS) for 6 h. Levels of pro‐inflammatory cytokines TNF‐α, CCL2, IL‐6, as well as anti‐inflammatory IL‐10, were analyzed by ELISA in the conditioned medium. Pre‐incubation with HG resulted in significant increased LPS‐induced M1‐related cytokines TNF‐α, CCL2 and IL‐6 in C57BL/6N PMΦ, compared to NG. In contrast, C57BL/6J PMΦ not only exhibited lower levels of pro‐inflammatory cytokines, but produced significantly more anti‐inflammatory IL‐10, indicative of a shift toward M2‐polarization. (d) Impact of NNT silencing on synergistic effect of HG and LPS. NNT silencing was performed in primary PMΦ from C57BL/6N mice, followed by treatment with different concentrations of glucose, additional LPS‐stimulation, and cytokine measurement as described under (c). Cells only treated with transfection medium served as controls (mock). Consistent with results in (**c**), mock control cells showed a significant increase in TNF‐α, CCL2, and IL‐6 after LPS‐stimulation, indicating M1‐polarization. Contrary to this, siNNT cells exhibited no significant increase in pro‐inflammatory cytokines after HG treatment and LPS‐stimulation, but instead, a strong increase in IL‐10, suggesting an important role in HG/LPS induced M1/M2 polarization. Naïve PMΦ were obtained from 5 to 6 female mice and pooled for each experiment. Data are presented as means ± SD from at least three independent experiments performed in triplicate for each condition.

Next, the effect of NNT knockdown on HG‐induced pro‐inflammatory CCL2 and pro‐fibrotic TGF‐β mediator release was investigated. Levels of CCL2 and TGF‐β were measured in the conditioned medium at 24 and 72 h, respectively. As expected, after incubating *Nnt*(+/+) cells with increasing glucose concentrations, both CCL2 and TGF‐β levels in conditioned medium were elevated after incubation compared to NG. NNT silencing significantly reduced CCL2 and completely abrogated TGF‐β release from MPMC cultured under HG conditions compared to mock control cells (Figure 5a). To investigate the possible role of NNT in MMT, MPMC were cultured under NG or HG conditions or stimulated with 10 ng/mL human recombinant TGF‐β for 24 h. Subsequently, α‐SMA, a marker for MMT, was evaluated. Up‐regulation of α‐SMA mRNA expression was observed after 24 h in both HG‐ and TGF‐β‐stimulated cells. At the protein level, up‐regulation of α‐SMA was detected only in MPMC stimulated with TGF‐β. NNT silencing did not affect MMT of MPMC in vitro (Figure 5b).

### 3.5 HG‐treated primary peritoneal macrophages exhibit increased susceptibility to LPS‐induced M1 polarization only in the presence of NNT

Previously, we (Balzer et al., 2019) and others (Grosick et al., 2018; Pavlou et al., 2018) have demonstrated that macrophages primed with HG are more susceptible to subsequent lipopolysaccharide (LPS)‐induced M1 polarization. To investigate a possible effect of genetic backgrounds on this response, we compared PMΦ from C57BL/6N and C57BL/6J mice. The naïve PMΦ were obtained via peritoneal lavage from healthy untreated mice, cultured under NG or HG conditions for 48 h, and thereafter stimulated with 100 ng/mL LPS for 6 h. Conditioned medium was analyzed for pro‐inflammatory (TNF‐α, IL‐6, CCL2) and anti‐inflammatory (IL‐10) mediators (Figure 5c). Under NG, no differences were observed between C57BL/6N and C57BL/6J PMΦ regarding LPS‐induced TNF‐α, IL‐6 and IL‐10 release. However, LPS‐induced CCL2 release was lower in C57BL/6J PMΦ compared to C57BL/6N PMΦ even under NG conditions. Culturing C57BL/6N PMΦ under HG conditions strongly increased LPS‐induced release of TNF‐α, IL‐6, and CCL2 compared to NG conditions. Additionally, a decreased level of IL‐10 was demonstrated under HG conditions. In contrast, PMΦ derived from C57BL/6J mice displayed a significant increase of IL‐10 under HG conditions compared to NG conditions. Moreover, LPS‐induced release of TNF‐α and CCL2 was not further enhanced by pre‐incubation with HG, and the HG effect on LPS‐induced IL‐6 release was less pronounced compared to C57BL/6N PMΦ. These findings indicate that HG‐treated C57BL/6N PMΦ are more prone to LPS‐induced M1 polarization than HG‐treated C57BL/6J PMΦ.

We next applied NNT siRNA knockdown in C57BL/6N PMΦ. We observed enhanced M1 polarization in mock control cells after HG pre‐incubation for 48 h followed by additional LPS stimulation for 6 h (Figure 5d). However, siNNT cells displayed no shift toward M1 polarization post‐LPS, phenocopying the effect observed in C57BL/6J mice. This highlights NNT's role in HG/LPS induced M1 polarization in peritoneal macrophages.

### 3.6 The reverse mode of action of NNT under HG conditions reduces NADPH level, increases mitochondrial ROS accumulation, and mediates HG‐induced cell cytotoxicity in NIH‐3 T3 fibroblasts

**FIGURE 6:**
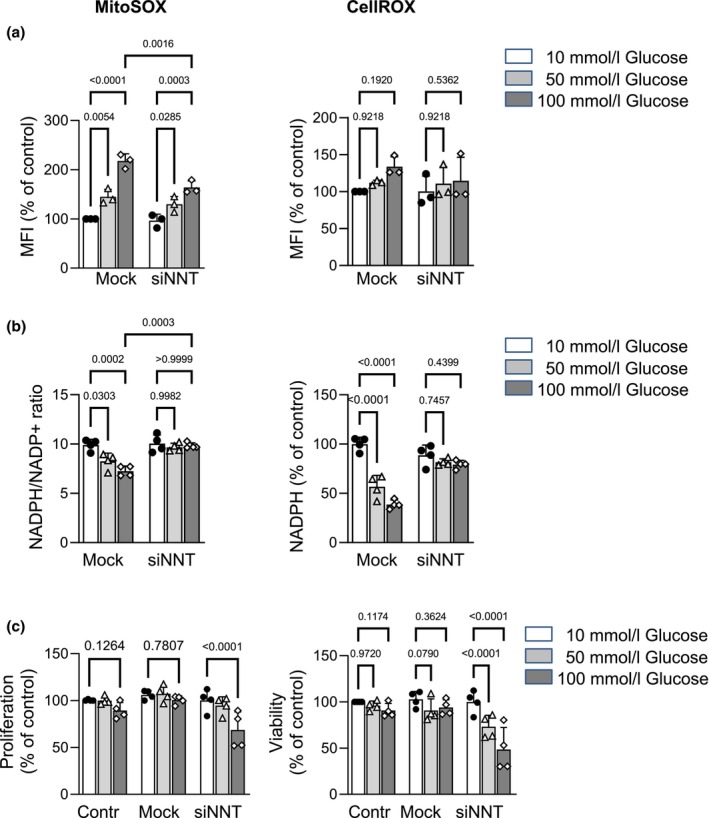
Effects of NNT silencing on HG‐induced responses in NIH‐3 T3 fibroblasts. (a) HG‐induced mitochondrial and cellular ROS accumulation was evaluated in NIH‐3 T3 fibroblasts after 24 h of incubation under HG conditions by FACS analysis using MitoSOX™ and CellROX™ kits, respectively. NNT knockdown resulted in significantly reduced mitochondrial ROS accumulation under HG conditions compared to mock control cells. This effect of NNT knockdown was highly specific for the mitochondrial compartment, as cellular levels of ROS measured by the CellROX staining were not affected. MFI = mean fluorescence intensity (b) The NADP(H) redox state under HG conditions was investigated using Promega® NADP/NADPH‐Glo Assay. NADPH/NADP^+^ ratio was significantly reduced in untreated and mock control cells after 24 h cultivation under HG conditions. This effect was completely abrogated in siNNT cells, suggesting a reverse mode of action in NNT under HG conditions. (c) Proliferation rate was measured using a CCK‐8 assay, after incubating cells in ascending glucose concentrations in presence of 10% FCS for 24 h. Glucotoxicity was evaluated after arresting cell proliferation by starving cells in 1% FCS, followed by treatment with varying glucose concentrations and subsequently measuring viability by the use of CCK‐8 assay. Untreated and mock control cells showed unaffected proliferation rates and cell viability under HG conditions, whereas siNNT cells exhibited a decreased proliferation rate after incubation in 100 mmol/L glucose. A significant increase of glucotoxicity was observed already at 50 mmol/L glucose. Data are presented as means ± SD from three independent experiments, performed in triplicate for each readout/condition.

Resident peritoneal fibroblasts are recognized as a source of peritoneal accumulating pro‐fibrotic myofibroblasts (Parikova et al., 2020; Witowski et al., 2015) and drivers of peritoneal damage (Okada et al., 2003). Since HG treatment has been shown to induce ROS‐mediated production of collagen and pro‐inflammatory cytokines in fibroblasts (Yin, 2018; Zhang et al., 2018), we assessed the role of NNT in HG‐induced responses in NIH‐3T3 fibroblasts. We stained control and siNNT cells with MitoSOX™ red and CellROX™ green to detect mitochondrial and cellular ROS, respectively. As expected, incubation of mock control cells with HG significantly increased mitochondrial and cellular ROS levels. NNT knockdown led to significantly reduced mitochondrial ROS accumulation under HG conditions. This effect of NNT knockdown was highly specific to the mitochondrial compartment, since the HG‐induced increase in cellular ROS levels remained unaffected (Figure 6a). These results challenge the conventional understanding of NNT as the main supplier of NADPH and key antioxidant enzyme in mitochondria (Kampjut & Sazanov, 2019; Ronchi et al., 2016; Rydstrom, 2006). Instead, our findings suggest that NNT may function in a reverse mode under HG conditions, resulting in NADPH oxidation (Nickel et al., 2015) and thereby reducing mitochondrial ROS levels after NNT knockdown.

To address this question, we measured NAD(P)^+^ and NAD(P)H levels in control and siNNT cells after 24 h of incubation with increasing glucose concentrations. The NADPH/NADP^+^ ratio was significantly reduced in untreated and mock control cells under HG conditions due to the decreased amount of NADPH. However, this HG‐induced effect was completely abrogated after NNT silencing; neither the amount of NADPH nor the NADPH/NADP^+^ ratio changed under HG conditions (Figure 6b), suggesting that under HG conditions, NNT indeed operates in reverse mode, consuming NADPH rather than generating it.

Given that HG‐induced cell cytotoxicity is primarily associated with oxidative stress (Buranasin et al., 2018), we further explored its effects on proliferation rate and cell viability. Cells were incubated in medium with increasing glucose concentration in the presence of 10% FCS for 24 h and thereafter, the proliferation rate was measured using the CCK‐8 assay. HG had no effect on the proliferation rate in untreated and mock control cells, whereas a decreased proliferation rate was observed in siNNT cells incubated with 100 mmol/L glucose (Figure 6c, left panel). To evaluate glucotoxicity, cell proliferation was arrested by decreasing the FCS concentration in the cell culture medium to 1%. While no glucotoxicity was observed in untreated and mock control cells, a significant increase in glucotoxicity was observed in siNNT cells already at 50 mmol/L glucose (Figure 6c, right panel).

These in vitro findings demonstrate that the reverse NNT reaction under extremely high glucose conditions contributes to mitochondrial ROS accumulation and promotes HG‐induced cellular responses. The prevention of the reverse NNT reaction may explain the reduced PM damage observed in C57BL/6J mice subjected to PD.

## 4 DISCUSSION

Accumulating evidence demonstrates phenotypic differences in behavioral, neurological, cardiovascular, and metabolic traits between the two most commonly used C57BL/6 sub‐strains C57BL/6J and C57BL/6N mice (reviewed in (Mekada & Yoshiki, 2021)). In the present study, we compared PD‐induced PM damage in these sub‐strains, revealing novel phenotypic differences. We observed that C57BL/6J mice were markedly protected from PD‐induced PM damage, characterized by preserved ultrafiltration capacity and reduced submesothelial fibrosis, inflammation, and neovascularization (Figures 1 and 2). Additionally, PD‐induced M1 polarization of PMΦ was not detected in C57BL/6J mice (Figure 3). These findings corroborate our previous findings highlighting the importance of genetic background in peritoneal fibrosis development in mice (Balzer et al., 2020; Helmke et al., 2019; Wang et al., 2016).

C57BL/6J mice, harboring a spontaneous loss‐of‐function deletion within the Nnt gene (Mekada et al., 2009; Simon et al., 2013), represent the only available model and a crucial tool for understanding the role of NNT in vivo. Impaired glucose tolerance in C57BL/6J mice as a result of glucose‐induced oxidative stress and decreased ATP synthesis in pancreatic beta cells due to the Nnt mutation has been reported (Toye et al., 2005). Furthermore, transgenic expression of the functional Nnt gene in mice with a C57BL/6J background resulted in improvements in glucose tolerance (Freeman et al., 2006; Kierans & Taylor, 2021). In inbred mouse strains of diabetes‐susceptible DBA/2xC57BL/6 backcrossed mice, a positive correlation between insulin secretion and NNT activity has been observed (Aston‐Mourney et al., 2007), although the forward/reverse NNT reaction was not investigated in these studies.

Since high glucose concentrations required for peritoneal dialysis significantly contribute to PD‐induced PM damage, and submesothelial NNT expression was strongly increased in PD‐treated C57BL/6N mice (Figure 4b), we hypothesized that NNT may mediate the adverse peritoneal changes induced by PD.

Although the results of in vivo experiments in this study were very conclusive, this model has inherent limitations, as the observed effects cannot be attributed solely to NNT deficiency. Besides NNT the C57BL/6N and C57BL/6J substrains exhibit several genetic variations encompassing SNPs, small insertions and deletions (indels), and structural variants (Simon et al., 2013) which may also contribute to PM damage. We previously demonstrated that PD‐induced peritoneal membrane damage is much less pronounced in SV129 mice compared to C57BL/6N mice (Wang et al., 2016) although both strains express wild type NNT.

To exclude other genetic influences and to directly elucidate the role of NNT, we performed in vitro experiments using an NNT‐specific siRNA approach to inhibit NNT expression in MPMC, PMΦ and NIH‐3T3 fibroblasts. These three cell types are known to play a pivotal role in PD‐induced peritoneal damage. Firstly, our results demonstrate that HG dose‐dependently upregulates NNT expression in MPMC and fibroblasts (Figure 4d).

MPMC promotes peritoneal deterioration via release of pro‐inflammatory and pro‐fibrotic mediators (Aroeira et al., 2005; Lee et al., 2012; Wang et al., 2016), and their mesothelial‐to‐mesenchymal transition compromises the mesothelial barrier severely. They are recognized as one of the main origins of ECM‐producing myofibroblasts (Padwal & Margetts, 2016). In MPMC, NNT knockdown resulted in decreased production of CCL2 and TGF‐β release under HG conditions (Figure 5a). However, NNT silencing did not affect HG‐ or TGF‐β‐induced MMT of MPMC, as indicated by unchanged α‐SMA expression (Figure 5b).

Resident peritoneal fibroblasts also contribute significantly to PD‐induced peritoneal damage, as they are considered another source of myofibroblasts (Witowski et al., 2015), exhibiting pronounced chemo‐tactic and pro‐inflammatory characteristics and increased proliferation (Kendall & Feghali‐Bostwick, 2014; Zhang et al., 2021). In our experiments, NNT silencing in fibroblasts resulted in significantly reduced cell viability and proliferation rate under HG conditions (Figure 6c). These results are in line with previously published observations made for SK‐Hep1 (Simon et al., 2013) and NCI‐H295R cancer cells (Chortis et al., 2018).

Macrophage M1/M2 polarization also contributes to peritoneal impairment (Li et al., 2018). Furthermore, enhanced glycolysis and a disrupted TCA cycle are associated with M1 polarization (Wculek et al., 2023). In line with this, we previously demonstrated that pre‐incubation with HG increased LPS‐induced M1 polarization in mouse PMΦ and in human macrophages (Balzer et al., 2019), while a similar priming effect has been shown for THP‐1 macrophages as well (Grosick et al., 2018). In this present study, LPS‐induced M1 polarization in naïve PMΦ from C57BL/6N mice under NG conditions was not affected by NNT‐knockdown; however, after pre‐incubation with HG, silencing of NNT prevented M1 defining cytokine release. These findings of siNNT experiments interestingly mirror in vivo results, as similar outcomes were observed in PMΦ of NNT‐silenced C57BL/6N and *Nnt*(−/−) C57BL/6J mice (Figure 5).

Extremely high glucose concentrations in PD solutions lead to increased peritoneal glucose absorption, resulting in a heightened cytosolic NADH/NAD^+^ ratio, a condition known as pseudohypoxia (Krediet, 2021, 2022). This metabolic state, characterized by elevated TGF‐β and hypoxia‐inducible factor 1 subunit alpha (HIF‐1α) expression (Jiang et al., 2015), enhances glycolysis and pyruvate production. HIF‐1α also inhibits the pyruvate dehydrogenase (PDH) complex and upregulates lactate dehydrogenase (LDHA), thereby diverting pyruvate away from the TCA cycle by decreasing acetyl CoA generation (Kierans & Taylor, 2021). Furthermore, HIF‐1α activation reduces alpha‐ketoglutarate dehydrogenase (α‐KGDH) activity, thus limiting glutamine‐dependent activity of the TCA cycle under PD conditions (Sun & Denko, 2014). Beta‐oxidation of fatty acids (FAO), another acetyl‐CoA source for the TCA cycle (Nolfi‐Donegan et al., 2020), has been demonstrated to decrease under PD conditions both in vitro and in vivo (Su et al., 2023). Consequently, all three primary energy substrates for the TCA cycle (glucose, fatty acids, and α‐KG) are limited under PD conditions, resulting in a state of low availability of mitochondrial respiratory substrates. Under conditions of insufficient NADH supply (Williams et al., 2021) or a malfunction of the electron transport chain, mitochondrial dysfunction can occur, possibly shifting the NNT reaction toward NAD^+^ reduction and NADPH oxidation (i.e., the reverse reaction), it can be postulated.

Our results indicate that under HG conditions, NNT does not serve as a protective mechanism against HG‐induced ROS generation and mitochondrial dysfunction. Vice versa, *Nnt*(+/+) C57BL/6N mice display aggravated HG‐induced PM damage in vivo and mediate pro‐inflammatory HG‐induced cellular responses in vitro. Therefore, we hypothesized that NNT might act in reverse mode under extremely high glucose concentrations. To assess this, we chose NIH‐3T3 fibroblasts, as these cells are very sensitive to HG (Buranasin et al., 2018) and exhibited the highest expression levels of NNT in our in vitro experiments. We observed significantly elevated mitochondrial ROS levels and significantly reduced NADPH/NADP^+^ ratios in NIH‐3T3 fibroblasts cultured under HG conditions compared to NG conditions, supporting the reverse NNT activity hypothesis under HG (Figure 6a,b). Notably, NNT silencing abrogated these HG‐induced effects. Although mitochondrial ROS was reduced, NNT silencing resulted in decreased cell viability and proliferation under HG conditions (Figure 6c), consistent with findings in rat aortic smooth muscle cells (Lopez‐Acosta et al., 2018). Here, the proliferation of these myofibroblastic cells was associated with both cellular and mitochondrial ROS release, ultimately leading to cell proliferation via secretion of SOFX (secreted oxidative stress‐induced factors) and subsequent activation of JACK/Akt/ERK1/2 pathways. Our in vivo findings are limited to NNT deficiency but cannot exclude other genetic differences between C57BL/6N and C57BL/6J substrains. Their potential role in PD‐induced PM injury remains to be investigated.

In summary, our study underscores the importance of genetic backgrounds in determining PD‐associated peritoneal inflammation and fibrosis by identifying a marked difference in susceptibility in two closely related mouse strains. Our in vitro and in vivo data strongly support the concept that the mitochondrial enzyme NNT plays a crucial role in the pathophysiology of peritoneal membrane dysfunction induced by PDF in C57BL/6N mice, and contributes to the phenotypic differences between the C57BL/6N and C57BL/6J substrains. We identify NNT as a potential therapeutic target to improve PD longevity in patients.

## AUTHOR CONTRIBUTIONS

**Nelli Shushakova**, **Yulia Kiyan**, and **Hermann Haller**: Study design. **Margarete C. Ohse**, **Song Rong, Sonja Schmidt**, **Nelli Shushakova**, **Yulia Kiyan**, and **Michael S. Balzer**: Experimental work. **Margarete C. Ohse**, **Nelli Shushakova**, **Yulia Kiyan**, **Michael S. Balzer**, and **Sibylle von Vietinghoff**: Data analysis. Manuscript preparation: All authors have approved the final version of the manuscript.

## FUNDING INFORMATION

This research was supported by grants to Michael S. Balzer from the German Research Foundation (Deutsche Forschungsgemeinschaft, DFG) (Ba 6205/1‐1). Parts of this manuscript have been presented in abstract form at ASN Kidney Week 2024. We thank Herle Chlebusch, Yvonne Nicolai, Michaela Beese, and Martina Flechsig for excellent technical assistance.

## CONFLICT OF INTEREST STATEMENT

All the authors declared no competing interests.

## ETHICS STATEMENT

The Department for Food Safety and Animal Welfare of Lower Saxony, LAVES, Germany (approval number: 33.19‐42502‐04‐16/2266) permitted the experiments.

## Supporting information


**Figure S1.** Uncropped gels for Figure 4c.
**Figure S2.** Uncropped gels for Figure 5b.
**Table S1.** Ohse et al.

## Data Availability

Primary data of this work are deposited at https://mhh‐publikationsserver.gbv.de/receive/mhh_mods_00003071and are available upon request.
