# Supplementary material for: Nicotinamide nucleotide transhydrogenase deficiency and genetic susceptibility to high glucose‐mediated peritoneal injury in mice
Source: Physiol Rep. 2025 Nov 14;13(22):e70642. doi: 10.14814/phy2.70642 (PMC12618214; doi:10.14814/phy2.70642)
Supplement: Supplementary file 1 — Figure S1. Uncropped gels for Figure 4c. Figure S2. Uncropped gels for Figure 5b. Table S1. Ohse et al. [file PHY2-13-e70642-s001.docx]

Supplementary – Ohse et al.

Table 1

| *Nnt* Primer  for allele-specific RT-PCR (ASQ) | Eurofins Scientific SE, Luxembourg, Luxembourg | Common GTA GGG CCA ACT GTT TCT GCA TGA  Wt fw. GGG CAT AGG AAG CAA ATA CCA AGT TG  Mutant fw. GTG GAA TTC CGC TGA GAG AAC TCT T |
| --- | --- | --- |
| *Ccl2* Primer | Eurofins Scientific SE, Luxembourg, Luxembourg | Fw. TGTGACTCGGACTGTGATGCCTTA  Rev. CTTGGAATCTCAAACACAAAGTTTACCC |

Supplementary figure 1. Uncropped gels for Figure 4 c

Supplementary figure 2. Uncropped gels for Figure 5 b
